# Guidelines for Accurate Multi-Temporal Model Registration of 3D Scanned Objects

**DOI:** 10.3390/jimaging9020043

**Published:** 2023-02-14

**Authors:** Kate J. Benfield, Dylan E. Burruel, Trevor J. Lujan

**Affiliations:** 1 Biomedical Engineering Doctoral Program, Boise State University, Boise, ID 83725, USA; 2 Department of Mechanical and Biomedical Engineering, Boise State University, Boise, ID 83725, USA

**Keywords:** structured light 3D scanning, mechanical wear, human tibia, knee, image registration, registration error, feature matching, iterative closest point, fiducial marker

## Abstract

Changes in object morphology can be quantified using 3D optical scanning to generate 3D models of an object at different time points. This process requires registration techniques that align target and reference 3D models using mapping functions based on common object features that are unaltered over time. The goal of this study was to determine guidelines when selecting these localized features to ensure robust and accurate 3D model registration. For this study, an object of interest (tibia bone replica) was 3D scanned at multiple time points, and the acquired 3D models were aligned using a simple cubic registration block attached to the object. The size of the registration block and the number of planar block surfaces selected to calculate the mapping functions used for 3D model registration were varied. Registration error was then calculated as the average linear surface variation between the target and reference tibial plateau surfaces. We obtained very low target registration errors when selecting block features with an area equivalent to at least 4% of the scanning field of view. Additionally, we found that at least two orthogonal surfaces should be selected to minimize registration error. Therefore, when registering 3D models to measure multi-temporal morphological change (e.g., mechanical wear), we recommend selecting multiplanar features that account for at least 4% of the scanning field of view. For the first time, this study has provided guidelines for selecting localized object features that can provide accurate 3D model registration for 3D scanned objects.

## 1. Introduction

In recent decades, there have been rapid advancements in the field of three-dimensional (3D) scanning, with applications in product inspection, reverse engineering, medical research, archeology, and construction of urban environment scenes [1,2,3,4,5,6]. Data collected from 3D scanners are used to generate 3D model reconstructions of an object of interest that can be used to evaluate geometric or dimensional object quality [4,7] and to quantify morphological changes [8], including mechanical wear in biomaterials [9,10,11]. Such quantification requires the registration of two separate 3D models. Registration is the process of spatially aligning two volumetric objects by transforming the objects into a common reference system [5], thus permitting the direct comparison of the superimposed 3D model surfaces and volumes. For this reason, accurate model registration is critical to measuring morphological differences between 3D scanned objects.

A standard step in 3D model registration is feature matching using maximum overlap techniques [5,12]. Maximum overlap aligns two 3D models by finding the optimal transformation between features on a reference and target 3D model and is most commonly achieved through Iterative Closest Point (ICP) algorithms [7,13] or variants of ICP [14,15]. These algorithms are generally applied to the entire 3D model geometry, such as when analyzing tolerances in quality part inspection [2]. However, there are drawbacks to selecting the entire 3D model geometry when detecting changes in localized morphology at multiple timepoints (e.g., mechanical wear and degradation), since these changes can create natural variations between the reference and target 3D models that can skew the mapping functions and cause registration errors [8].

An alternative to selecting the entire 3D model geometry when optimizing object registration is to instead select only a subset of the 3D model that contains distinct geometric features that are unaltered over time [12,16]. These unaltered regions can be used to optimize the mapping functions between the reference and target 3D models, and then, the optimized transformation can be applied to the entire object. This allows any altered regions between the target and reference objects to not influence 3D model registration and ultimately permits any real morphological differences between objects to be accurately quantified. Many studies have used this approach for multi-temporal 3D model registration by selecting a wide variety of features. These features have consisted of either intrinsic features (natural to the object) [8,9,11,17] or artificial features (intentionally placed marker) [4,18,19]. For example, Valigi et al. evaluated wear in an artificial knee by selecting unworn portions of a tibial component to register knee models that were generated at different time points (i.e., pre-wear and post-wear) [11]. More recently, we measured in vitro fibrocartilage wear in the human knee by selecting artificial features fastened to the tibia bone to register 3D models before and after joint loading to simulate pre- and post-wear time points [19]. The accurate application of this common registration method is dependent on the local artificial or intrinsic features selected by the operator to calculate the registration transform, yet there are limited guidelines for selecting these features. This lack of guidance has resulted in wide variability in the sizes and shapes of features used to register 3D models. For example, studies by Hawryluk et al. and Verhoeven et al. used intrinsic features that were approximately 30–70% the total object surface area [8,17], while Helle et al. and Seminati et al. used artificial features that were roughly 100% and 1% the size of the object surface area, respectively [4,18]. Furthermore, planar geometry of selected features is not well understood. While Yao et al. determined that using planar features instead of linear features for registration increased registration performance by nearly 50% [20], the number of orthogonal planes necessary to optimize the registration was not specified. Therefore, there is a lack in current understanding for how selected feature geometry (e.g., size and shape) influences overall registration accuracy. This information is needed to ensure the quality and reproducibility of morphological changes measured from 3D models.

The objective of this study was to determine practical guidelines for selecting object features when registering 3D models at different time points (multi-temporal) by measuring the effect of selected feature geometry on overall registration accuracy. We conducted this analysis using simple cubic registration blocks to register complex 3D models of a tibia bone replica that we acquired at multiple time points with 3D scanning.

## 2. Materials and Methods

### 2.1. Registration Block and Object of Interest

**Figure 1:**
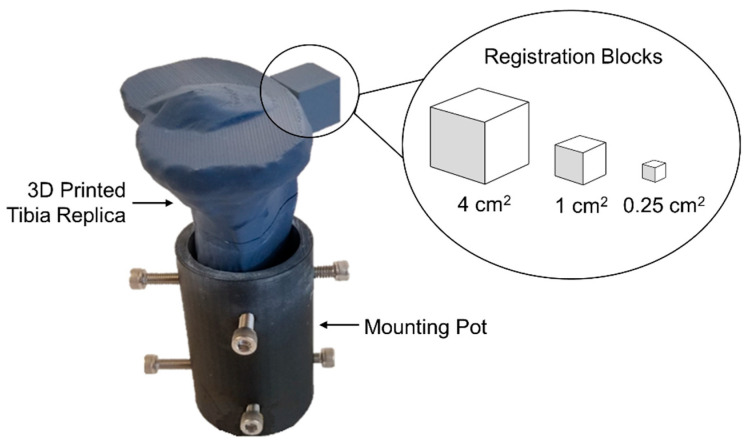
Three-dimensional printed tibia replica within a mounting pot showing the placement of the cubic registration block. Three separate tibia replicas were printed with each block size (cross-sectional area of cube face = 4, 1, and 0.25 cm^2^).

A simple cube was selected to serve as the registration block, as it is an easily replicable geometric primitive. A total of three blocks were designed in SolidWorks (Waltham, MA, USA; v2021) with varying sizes of large, medium, and small corresponding to cross-sectional areas (CSA) of 4, 1, and 0.25 cm^2^, respectively. These sizes were selected based on a series of pilot tests that found registration error to vary considerably between 0.25 and 4 cm^2^. These pilot tests also showed that increasing the block size above 4 cm^2^ had a negligible effect on the registration error. A replica of a human tibia bone was selected as the object of interest [19]. Within modeling software (Meshmixer, Autodesk, v3.5), three separate 3D models of the tibia bone geometry and the 3D registration blocks were imported as object files. Each block was then separately attached to the anterior-central side of the tibia (Figure 1). This resulted in three total 3D tibia geometries that were then 3D printed using a fused deposited modeling (FDM) printer (Pursa Research, MKS3; z resolution ±50 μm; fine detail; 15% infill). Matte blue polylactic acid (PLA) filament (Hatchbox) was used to improve the scan quality by minimizing light reflectance.

### 2.2. 3D Scanning and Model Registration

**Figure 2:**
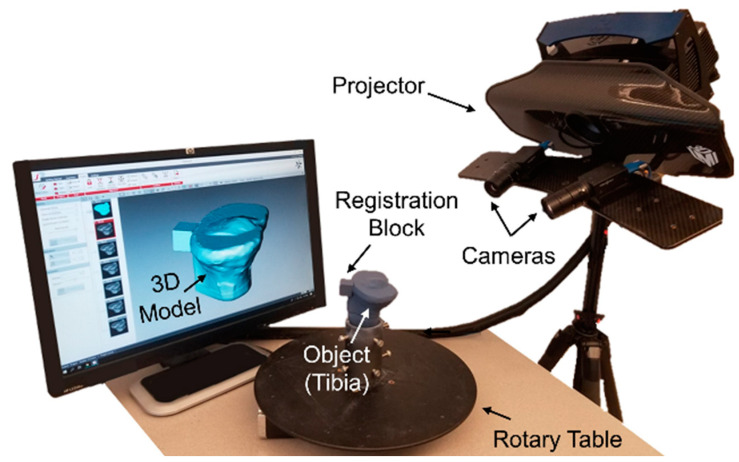
The 3D optical scanner. The projector emits a light pattern upon a rotated object of interest (tibia), while two cameras record the distortion of the projected light. The scanning software then converts these data into surface coordinates to create a 3D model.

The 3D optical scanning system consisted of a rotary table, projector (HDI Advance R3 projector with 16 mm lenses), and two high-resolution cameras (LMI Technologies, Delta, Canada; 2.8 megapixels) (Figure 2). The scanning system used FlexScan3D software (LMI Technologies, Delta, Canada; v3.3.21.8), and the system was calibrated using a calibration card consisting of a 6 × 4.5 cm grid of 5 × 5 mm black and white squares [10]. The procedure to generate and register 3D models with the scanning system can be split into two steps: data acquisition and post-processing.

**Figure 3:**
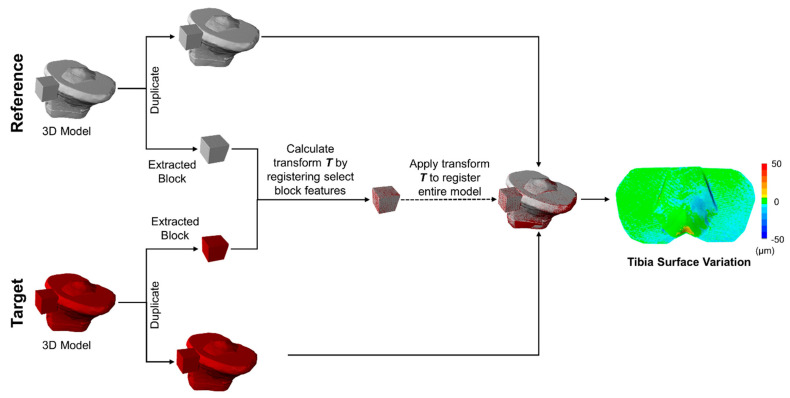
Flowchart of the post-processing methodology to measure surface variation. Three-dimensional models of target and reference objects were registered, and the linear surface variation was recorded as the mean deviation between the registered objects. Registration of the whole object (tibia) used the transformation matrix computed between the manually extracted registration blocks of the target and reference 3D models.

For data acquisition, the tibia was mounted in a pot (Figure 1) and centered on the rotary table (Figure 2). The 3D scanner was used to generate two identical 3D models to serve as the reference and target following the experimental method (Figure 3). All scanning was performed in a dark room, with the only light source coming from the projector [10,19]. This setting was used to minimize the presence of ambient light that could impact scanning quality. The projector field of view (FOV) was 200 cm^2^ (resolution = 0.12 mm). The exposure settings were set to high dynamic range (HDR), which automatically determined the optimal exposure (range of exposure times = 16.67–150 ms) [19]. The tibia was scanned every 30 degrees using the rotary table (Figure 2) for a total of 12 scans, resulting in a full 360-degree captured view. For each block size (4, 1, and 0.25 cm^2^), only one reference 3D model was generated, while the target 3D model was generated in triplicate, resulting in 12 total 3D models. The entire data-acquisition step took approximately 10 min to complete per 3D model.

In post-processing, all 12 scans were combined into a 3D model by automatically fine aligning each scan based on common geometry within the commercial scanning software [10,19]. This was carried out to generate 3D models for the reference and target conditions. The target 3D models were registered (i.e., merged) to the 3D reference model using feature matching by selecting features on the fixed geometry of the registration block. To perform this step, each 3D model was duplicated, where the duplicate had all regions manually removed, except for the registration block (Figure 3). The extracted target and reference registration blocks were first roughly aligned by manually translating and rotating the objects and were then automatically finely aligned using an ICP-based function called *Mesh Geometry* within the scanning software. The transformation matrix T was automatically calculated and recorded during this registration process. This transformation matrix was then used to register the whole 3D reference model to the whole 3D target model (Figure 3). In total, this post-processing step took approximately 10 min to complete, with an average runtime for the automated model registration of 1.0 ± 0.5 s. The larger markers had an average registration runtime (1.56 s) more than double the runtime for smaller markers (0.61 s), while increasing the number of analyzed planes added on average less than 0.2 s to the registration runtime.

### 2.3. Selection of Cubic Registration Block Features (Planes)

**Figure 4:**
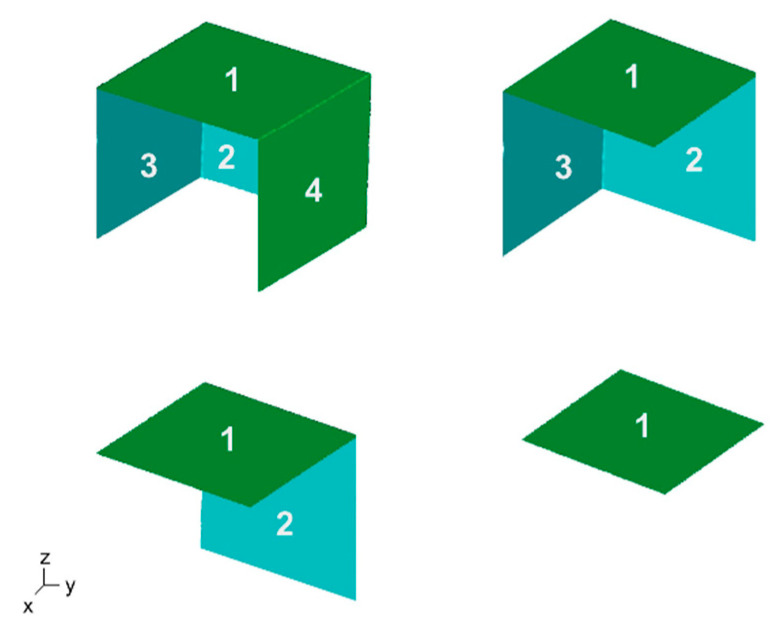
Variation in the number of selected planar features ranged from four planes to one plane.

To investigate the impact of registration block features on 3D model registration, the number of geometric planes on the block was varied (Figure 4; 1, 1-2, 1-2-3, and 1-2-3-4) for each block size. Open-sourced software (CloudCompare, Telecom, Paris, v2.10.3) was used to manually select and remove desired planes within the reference and target 3D models. The aforementioned 3D registration process (Section 2.2) was then repeated for each registration block size (*n* = 3) and number of planes (*n* = 4) in triplicate, resulting in 36 registered 3D models.

To determine whether different planar configurations would influence registration accuracy, we varied the configurations for the one-plane and two-plane cases on the large registration block. For the one-plane case, we evaluated the registration error using planar surfaces 1, 2, 3, and 4 separately. For the two-orthogonal-plane case, we assessed the following planar configurations: 1-2, 1-3, 1-4, 2-3, 2-4, and 3-4. The lowest registration errors occurred when using plane 1 for the one-plane case and planes 1-2 for the two-orthogonal-plane case (block errors of 9.8 ± 5.3 µm and 4.8 ± 0.9 µm, respectively), and the highest registration errors occurred when using plane 3 for the one-plane case and planes 3-4 for the two orthogonal plane case (block errors of 46.8 ± 41.2 µm and 25.4 ± 22.1 µm, respectively). However, any differences in the registration errors between the planar configurations for both the one-plane and two-plane cases were not significant (*p* > 0.05; one-way ANOVA).

### 2.4. Surface Variation Analysis

CloudCompare was used to extract the tibial plateau surface within the registered 3D models by applying a user-specified snipping pattern around the region of interest. The snipping pattern was identically applied to all 3D models, ensuring consistency between the tibial surface regions [19]. Within the scanning software (FlexScan3D), the average surface variation between the reference and target tibial plateau surfaces was then calculated. It is important to note that the registration of identical reference and target 3D models should yield surface variations of zero; therefore, any deviation from zero is likely due to a registration error. For this reason, registration error was quantified as the linear surface variations on the tibial surface.

### 2.5. Statistical Analysis

Statistical software SPSS (IBM v26.0) was utilized for all data analysis. The effect of registration block size (4, 1, and 0.25 cm^2^) and number of planes (4, 3, 2, 1) on the tibial surface variation (registration error) was assessed using a repeated measures ANOVA. The number of planes was considered a within-subjects factor, while block size was a between-subjects factor. A similar repeated measures ANOVA was conducted to determine the effect of block size and number of planes on the surface variation measured on the cubic registration blocks themselves. A Bonferroni adjustment was used for pairwise comparisons. Significance was set at *p* < 0.05, and all data are reported as mean ± standard deviation.

## 3. Results

**Figure 5:**
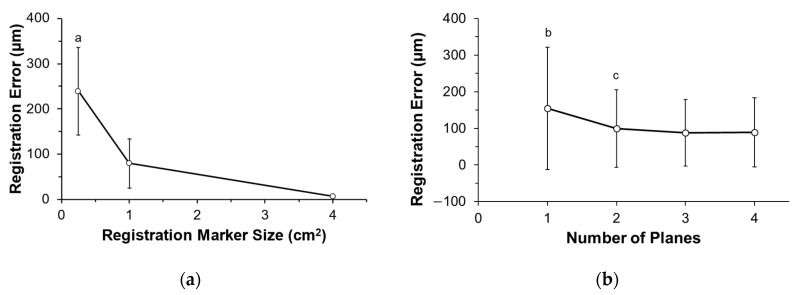
Average registration error on the tibia surface. (**a**) The largest block produced the lowest error, and (**b**) a plateau in variation occurred once at least two planes were used for registration. ^a^ = greater error than all larger sized block (*p* < 0.05), ^b^ = greater error than two, three, or four planes (*p* < 0.05), ^c^ = greater error than four planes (*p* < 0.05).

Average registration error on the tibial plateau surface was significantly influenced by the registration block size (*p* = 0.006). The lowest average error of 5.8 ± 3.4 µm was achieved using the largest block size with errors nearly 40x lower than that of the smallest block (*p* = 0.007) and 14x lower than the medium block (*p* = 0.492) (Figure 5a; Table 1). Moreover, the number of geometric planes selected for registration influenced the registration error on the tibial surface (*p* = 0.004). With only one plane selected, error was 55–75% greater than all other planar configurations (Figure 5b; *p* < 0.05). A significant interaction existed between the selected number of planes and the registration block size, where increasing the number of planes resulted in lower registration errors in larger block sizes (*p* = 0.013).

**Figure 6:**
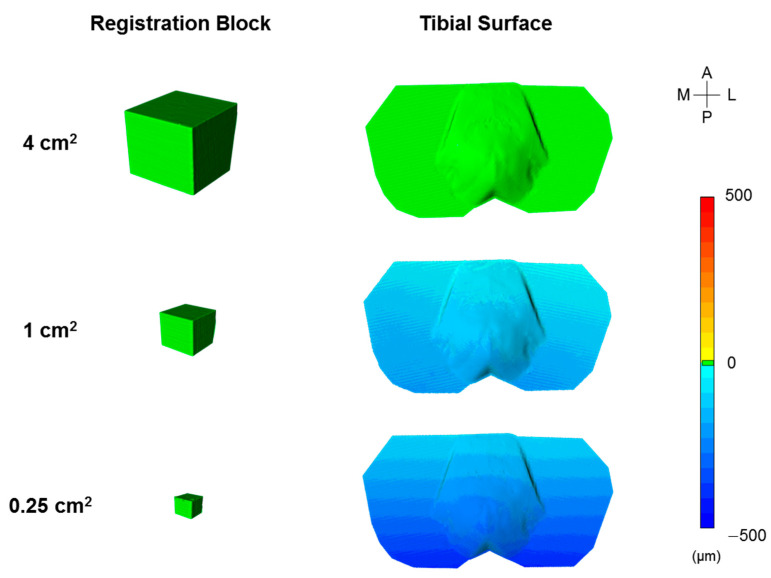
Linear surface variation after registering two identical objects (tibia) that were acquired using a 3D optical scanner. In this figure, 3D objects were registered using four planes of the registration block. The posterior region of the tibial plateau surface could not be accurately registered when using smaller registration blocks.

Qualitatively, the surface variations on the tibial plateau surface of the registered models were greater when using the smaller registration blocks, with a higher concentration of variation in the posterior aspects of the tibial surface (Figure 6; dark blue). The surface variation on the surfaces of the registration markers themselves were relatively consistent (Figure 6; green cubes), and quantitatively, the registration errors on the surfaces of the medium (9.1 ± 3.9 μm) and small (12.4 ± 1.4 μm) registration markers were not significantly different (*p* = 0.38). However, the registration error on the surface of the large (3.0 ± 1.6 μm) registration markers was significantly less than all other marker sizes (*p* < 0.05).

## 4. Discussion

The field of 3D scanning technology is rapidly advancing with numerous applications, yet accurate 3D model registration for certain applications remains a challenge (e.g., wear analysis). The goal of this study was to determine guidelines for accurate and robust model registration with 3D scanned objects. To the best of our knowledge, this is the first study to provide such guidelines.

A key finding from this study was that registration accuracy was dependent on the size and selected features (planes) of the registration block (Figure 5; *p* < 0.05). When using the largest block (CSA = 4 cm^2^) with two planar features, our registration error was 4.8 ± 0.9 µm (Table 1). As the size of the block decreased, a higher error propagated towards the posterior aspect of the tibial plateau surface away from the registration block (Figure 6; tibial surface, dark blue). This indicates that the surface area of the smaller blocks is insufficient to develop the transformation required to accurately register the whole reference and whole target models. Interestingly, the surface variations on the surfaces of the smaller registration blocks were relatively small (12.4 ± 1.4 μm). This relatively small error could lead to a false sense of security if using small artificial features to register objects, since the artificial features themselves would appear accurately registered, while the object of interest that is being analyzed would likely have poor registration accuracy. When determining the number of surface features (planes) that should be selected on the registration block to minimize error, we found a significant reduction in error when increasing the number of selected orthogonal planes from 1 to 2, with error plateauing when more planes were selected (Figure 5b). This was surprising as we expected increases in the number of planes and corresponding surface area to reduce registration error. These findings suggest that any natural or artificial geometries used for feature selection should have at least two orthogonal surfaces, but more than two orthogonal surfaces would provide only nominal improvement. Additionally, we found that selection of the planar configurations most aligned with the object surface being analyzed (i.e., plane 1 and orthogonal planes 1-2; Figure 4) resulted in the lowest average registration errors, although any changes in registration errors due to different planar configurations were not significant.

**Figure 7:**
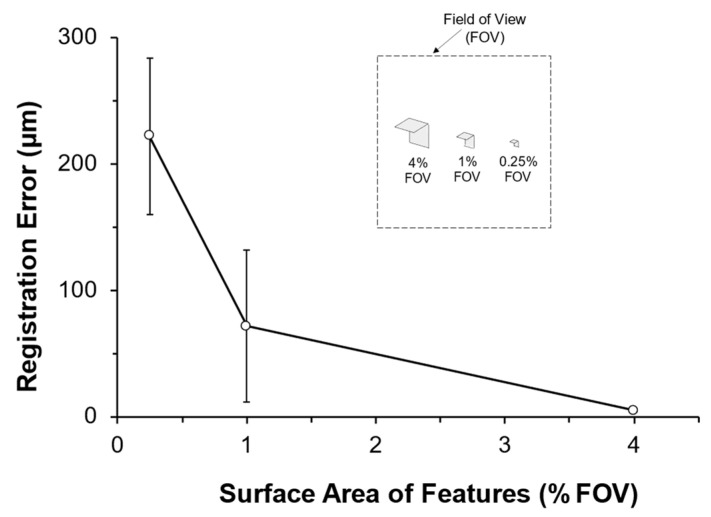
Registration error relative to the cross-sectional area of two selected features (planar surfaces) in terms of % field of view (% FOV). The small error when selecting block features accounting for 4% FOV is similar to the average error when using the entire 3D model (tibia + block; 96% FOV) to optimize the transformation.

Results from this study can be depicted in terms of the scanner’s field of view (FOV). This is carried out by normalizing the surface area of the selected block features used for 3D model registration to the cross-sectional area of the scanner’s FOV (% FOV) (Figure 7). This gives a simplistic way to determine the necessary size of the registration block to produce an accurate registration for a user-specified FOV. Using this approach, we see that blocks with features equivalent to 1% FOV resulted in registration errors upwards of 70 µm, while blocks with features equivalent to 4% FOV reduced errors to 4.8 ± 0.9 µm (Figure 7). Furthermore, when evaluating features from the large block corresponding to 6% FOV (three-plane case) and 8% FOV (four-plane case), the registration errors were 4.6 ± 1.5 µm and 4.1 ± 1.5 µm, respectively. These small errors are comparable (*p* = 0.067) to the average error of 2.9 ± 1.2 µm when using the entire 3D model (96% FOV) to optimize the mapping functions required for registration. This suggests that increasing the size of the registration block above 4% FOV would lead to only nominal improvements in registration accuracy. We should reiterate that for our study design, the object morphology of the reference and target 3D models was identical. Therefore, registration was optimized when using the entire object geometry to obtain the mapping function. However, when morphological differences do exist between the reference and target 3D model (e.g., mechanical wear), mapping functions obtained from an unaltered subset of the 3D model (i.e., registration block) can have significantly lower target registration errors compared to selecting the entire object geometry [10].

Our results can be compared to other studies that registered 3D models by selecting intrinsic (natural to object) or artificial (intentionally placed) features on their object of interest. A study by Hollar et al. used a 3D optical scanner (LMI Technologies; FlexScan3D; FOV = 20 cm^2^) to assess the mechanical wear within hip protheses mounted in a custom stand by registering pre- and post-wear 3D models [10]. The model was registered by selecting complex intrinsic features on the relatively large stand and resulted in a registration error of approximately 5.1 ± 1.0 µm, very close to our own results when using a large registration block (Table 1). Landi et al. used a portable 3D laser scanner (FreeScan UE7; Geomagic Control X; maximum FOV = 2652 cm^2^) to analyze the deformation of machine guards before and after ballistic penetration, where 3D models were registered using intrinsic features consisting of the undeformed edges of the guards, with registration errors of approximately 300 µm [21]. Based on the results of the present study (Figure 5b), this registration error may have been reduced if orthogonal surfaces were selected for registration rather than edges. A study by Seminati et al. applied hemispherical adhesives to a residual limb model to serve as fiducial markers during optical 3D scanning (Artec Eva; Artec Studio 9.2 Studio; maximum FOV = 1989 cm^2^), resulting in registration differences of less than 1000 µm [18]. Campanelli et al. adhered spherical markers directly to a bovine femur to evaluate the reliability of the 3D models produced by a 3D laser scanner (Creaform; Geomagic Studio; maximum FOV = 688 cm^2^), with a registration error of approximately 70 µm [22]. Collectively, these prior results from other groups show 15–200× higher error, with 3–13× greater FOV than reported in the present study. This demonstrates that a simple cubic registration block can provide equal to superior performance to other more complex feature geometries used for 3D model registration.

There are notable limitations to this study. First, all of our results were based on the evaluation of planar features extracted from a cubic registration block. Other geometries may have performed well; however, a cube is simplistic, easily replicable, and produced low target registration errors (Table 1). Second, we positioned our registration block relatively close to the tibial surface to minimize the distance between the registration block and surface being analyzed in order to enhance registration accuracy. Placement of the block further from the surface being analyzed would likely result in greater registration error, although the variability in the colorimetric map of the tibial surface would suggest that any increase in error would be relatively small when using the largest block size (Figure 6). Lastly, all tests were performed using one type of 3D optical scanner and accompanying software program. While other types of scanner systems (e.g., 3D laser scanners) may have produced different registration errors, we would expect that the relative changes we observed in registration errors when altering the selected features (Figure 5) would be consistent and that the conclusions from this study are applicable to other scanning systems that utilize ICP-based algorithms.

## 5. Conclusions

In summary, this study identified key criteria for utilizing localized features to register two 3D models. We determined that features with at least two orthogonal surfaces and with a surface area that is at least 4% of the scanning field of view will produce minimal registration errors. In addition, if using an artificial marker for feature matching registration, a primitive cube provides excellent registration accuracy. The results from this study provide a benchmark for future advancements in model registration, and the recommendations from this study can be used by other researchers, industry professionals, and 3D model enthusiasts to improve the accuracy and robustness of 3D model registration, especially when evaluating changes to object morphology (e.g., mechanical wear).

## Figures and Tables

**Table 1:** Average registration error (μm) on the tibial plateau surface for all block sizes and number of selected planes.

Registration Block Size	Number of Planes				
	4	3	2	1	Average
Large	4.1 ± 1.5	4.6 ± 1.5	4.8 ± 0.9	9.8 ± 5.3	5.8 ± 3.4
Medium	68.9 ± 57.3	69.6 ± 53.9	71.7 ± 60.0	106.5 ± 72.7	79.2 ± 54.9
Small	194.6 ± 66.9	191.1 ± 57.9	221.8 ± 62.0	346.7 ± 125.9	238.6 ± 97.0
All	89.2 ± 94.8	88.4 ± 91.0	99.4 ± 105.5	154.3 ± 166.9	107.8 ± 116.7

## Data Availability

The data presented in this study are available on request from the corresponding author.
